# Human iPSC for Therapeutic Approaches to the Nervous System: Present and Future Applications

**DOI:** 10.1155/2016/4869071

**Published:** 2015-11-30

**Authors:** Maria Giuseppina Cefalo, Andrea Carai, Evelina Miele, Agnese Po, Elisabetta Ferretti, Angela Mastronuzzi, Isabelle M. Germano

**Affiliations:** ^1^Department of Hematology/Oncology and Stem Cell Transplantation, Bambino Gesù Children's Hospital, IRCCS, Piazza Sant'Onofrio 4, 00165 Rome, Italy; ^2^Department of Neuroscience and Neurorehabilitation, Neurosurgery Unit, Bambino Gesù Children's Hospital, IRCCS, Piazza Sant'Onofrio 4, 00165 Rome, Italy; ^3^Department of Molecular Medicine, Sapienza University of Rome, 00161 Rome, Italy; ^4^Center for Life Nano Science @Sapienza, Italian Institute of Technology, 00161 Rome, Italy; ^5^Department of Experimental Medicine, Sapienza University of Rome, 00161 Rome, Italy; ^6^Department of Neurosurgery, Icahn School of Medicine at Mount Sinai, New York, NY 10029, USA

## Abstract

Many central nervous system (CNS) diseases including stroke, spinal cord injury (SCI), and brain tumors are a significant cause of worldwide morbidity/mortality and yet do not have satisfying treatments. Cell-based therapy to restore lost function or to carry new therapeutic genes is a promising new therapeutic approach, particularly after human iPSCs became available. However, efficient generation of footprint-free and xeno-free human iPSC is a prerequisite for their clinical use. In this paper, we will first summarize the current methodology to obtain footprint- and xeno-free human iPSC. We will then review the current iPSC applications in therapeutic approaches for CNS regeneration and their use as vectors to carry proapoptotic genes for brain tumors and review their applications for modelling of neurological diseases and formulating new therapeutic approaches. Available results will be summarized and compared. Finally, we will discuss current limitations precluding iPSC from being used on large scale for clinical applications and provide an overview of future areas of improvement. In conclusion, significant progress has occurred in deriving iPSC suitable for clinical use in the field of neurological diseases. Current efforts to overcome technical challenges, including reducing labour and cost, will hopefully expedite the integration of this technology in the clinical setting.

## 1. Introduction

Several diseases affecting the central nervous system (CNS) including stroke, spinal cord injury (SCI), and brain tumors remain the leading causes of mortality and morbidity in the US and worldwide [1]. Current therapies are still not fully successful in restoring the damaged tissue, in the case of stroke and SCI, or in selectively killing tumor cells dispersed in otherwise normal parenchyma, while sparing the latter, in the case of brain tumors. Cell-based therapies offer the potential advantages to provide regenerative tissue or to provide “vectors” aimed at targeting diseased cells. One additional challenge to improve therapies for CNS diseases is a better understanding of their pathophysiology, particularly for neurodegenerative diseases, such as Parkinson's diseases [2] or amyotrophic lateral sclerosis (ALS) [3]. For this purpose, information that can be derived from patient's specific cells offers a great tool to accelerate the understanding of mechanisms at the base of these conditions, possibly providing new therapeutic approaches.

The isolation of embryonic stem cells (ESC) was initially considered the most innovative strategy to approach “cell-based regenerative medicine” [4] due to their pluripotent nature, their unrestricted power of self-renewal, and their ability to autodifferentiate into any cellular type. Unfortunately, many aspects have limited their application in treating human diseases, including ethical and technical issues, such as their derivation from early-stage embryos and the immune rejection for nonautologous cell lines [5]. Subsequently, the elaboration of “nuclear cloning” [6] or mammalian somatic cell nuclear transfer seemed to solve some of these limitations by creating a cloned cell from which to isolate the nuclear transfer-derived ESC, as autologous donor cells for therapy. This strategy demonstrated feasibility in a mouse model of immunodeficiency [7] but was not successfully reproduced in humans.

In 2006, Takahashi and Yamanaka [8] developed a line of induced pluripotent stem cells (iPSCs) using fibroblasts. They identified 24 candidate genes highly expressed in ESC critical to confer and maintain pluripotency. These genes were introduced into the mouse fibroblasts by a retroviral vector, demonstrating the reprogramming of somatic cells back to an ESC-like pluripotent state. iPSCs were first induced by the transfer of only four genes [9], Oct4, Sox2, Klf4, and c-Myc. This approach was applied to adult human fibroblasts, leading to the creation of human iPSC [10]. Due to the potential genomic integration of transgenes resulting from the use of retroviruses containing the oncogene c-Myc, the original technique carried a significant risk of tumorigenesis. Recent improvements in nuclear reprogramming have made iPSC induction safer as genes transfer can be achieved with techniques other than viral transduction [11–14], thus eliminating the risk of genomic integration. This is clinically significant when iPSCs are considered for transplant, as they represent a promising tool for regenerative medicine, in pathologies such as cardiomyopathies [15], stroke [16], and SCI [17].

The main characteristic of iPSC is pluripotency [18], defining the ability to differentiate into three germ layers and all cell types. The advantage of patient-specific iPSC is twofold. In disease modelling, the effects of patient-relevant mutations can be studied in the correct genetic and cellular background. In cells-based therapy, patient-specific iPSC will obviate the needs of immune suppressors. The elaboration of disease-derived iPSC [19] was first obtained in 2008 from a patient with ALS. These patient-specific iPSCs were successfully directed to differentiate into motor neurons, representing a potentially novel platform for disease modelling. Advances in induction of patient-specific iPSCs allowed their use to model a widespread variety of patient-specific diseases, such as cardiomyopathies [16] and as recently reported chemotherapy induced neurotoxicity [20].

Finally, iPSC-derived cells can be used in cell-based therapy as vectors to carry genes to their original organ. This has been explored for brain tumors using ESC and NPC [21, 22]. The rationale for this approach relies on the fact that primary brain tumors are very aggressive, infiltrative, and invasive, thus requiring cell-based therapy that can target tumoral cells while sparing the normal brain [22].

To accomplish the goals of using iPSC in large scale, numerous technical advances need to be pursued including reducing labour and cost to produce iPSC in large scale. In 2009 in the US, the FDA approved the country's first human trial on ESC transplantation into patients suffering from SCI; the trial, however, came to a halt in November 2011 when the company financing the trial announced the discontinuation of the trial due to financial issues [23]. Additionally, iPSC should be “safe” and easily obtainable from body sources with minimal invasiveness and high efficiency of reprogramming, overcoming three major current obstacles. First, the risk of genomic modification due to viral transgenes needs to be overcome by insertion-free or “footprint-free” iPSC. Second, the risk of teratogenicity if undifferentiated iPSCs are engrafted requires full differentiation or reprogramming inactivation of iPSCs before transplant. Finally, the risks of transmission of nonhuman pathogens to humans and/or immune response concern triggered by contamination from nonhuman antigens, deriving from the xeno-cell-dependent culture systems, necessitate the development of xeno-free iPSCs. Techniques and results used to overcome these burdens are described below.

## 2. Methods

**Figure 1:**
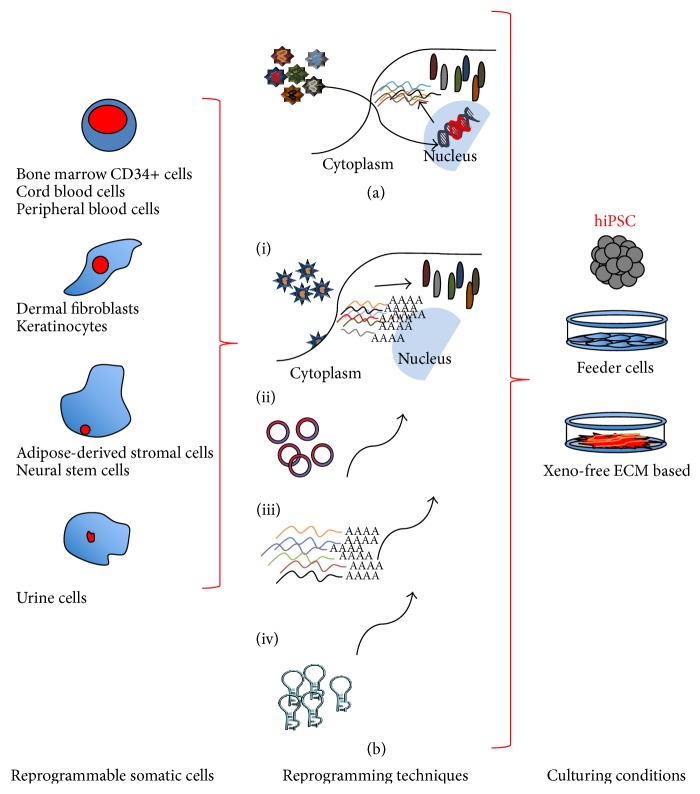
Diagrammatic representation of methods used to obtain human iPSC. Different somatic cells can be used for reprogramming (left column). Reprogramming techniques (center column) first used viral based genomic integration (a) and then used footprint-free techniques (b). Footprint-free iPSC induction can be obtained by Sendai virus (b(i)); episome (b(ii)); mRNA (b(iii)); siRNA (b(iv)). Finally, culturing conditions (right column) at first requiring feeder cells evolved to xeno-free conditions to allow safer clinical translation.

Figure 1 summarizes methods to obtain iPSC. Different somatic cells can be used for reprogramming (Figure 1, left column). Reprogramming techniques (Figure 1, center column) first used viral based genomic integration (Figure 1(a)) and then used footprint-free techniques (Figure 1(b)). Finally, culturing conditions (Figure 1, right column) at first requiring feeder cells evolved to xeno-free conditions to allow safer clinical translation. Methodologies summarized in the diagram are briefly reported below.

### 2.1. Reprogrammable Somatic Cells for iPSC

Ideally, cell sources of hiPSC should be acquired easily and noninvasively from patients and should be reprogrammed into iPSCs with high efficiency. Cell types successfully utilized for hIPSC production include dermal fibroblasts [10], bone marrow CD34+ cells [32], cord blood cells [33], peripheral blood cells [34], adipose-derived stromal cells [35], neural stem cells [36], and keratinocytes [37] (Figure 1, left column).

Recently, Nakagawa et al. [38] were able to obtain an adequate number of footprint-free, xeno-free hiPSC clones from both skin-derived fibroblasts and blood cells. Lee et al. [39] reported a method to generate footprint- and xeno-free iPSC from urine cells which can be obtained totally noninvasively using extracellular matrix-based xeno-free iPSC culture condition and episomal transfection.

### 2.2. “Footprint-Free” iPSC

The reprogramming of somatic cells to pluripotency implicates the risk of genomic modification when retroviral and lentiviral vectors are used. Indeed, although these vectors are feasible and efficient in iPSC production, they also cause insertional mutagenesis due to viral vector integration, prompting caution with their translation to clinical applications (Figure 1(a)).

Among the first attempts to produce footprint-free iPSC was the use of nonintegrating vectors encoding reprogramming factors (RF) based on adenovirus [40] and transient plasmid to be repeatedly transfected [41, 42]. However, this resulted in much lower reprogramming efficiency with still some residual risk of genomic alteration, thus necessitating PCR screening of iPSC colonies or sequencing before taking them forward to clinical application.

Another intriguing system is represented by episomes (Figure 1(b(ii))) [43, 44] where the expression vector is circular DNA encoding RF that is incorporated by cells through penetrating peptide moieties in culture media. The episomes show rapid and persistent RF expression, allowing a single transfection procedure to obtain iPSC, while they are lost by dilution over several weeks [45]. Nonetheless, episome-derived iPSCs need to be checked for genomic recombination and successful clearance of the RF, making their clinical applicability far from optimal.

Later on, new attracting methods to generate footprint-free iPSCs with higher reprogramming efficiency were developed: the RNA virus (Figure 1(b(i))), Sendai virus (SeV) [46], and mRNA or modified RNA (modRNA) [47] (Figure 1(b(iii))). In the SeV, RNA system RF are infected into cells by using a recombinant animal virus with a completely RNA-based replication cycle. Robust iPSC colonies are generated in 2-3 weeks, with efficiency even higher than the conventional retroviral and lentiviral protocols. As with the episomal method, the SeV RNA has the “one-shot” advantage and is lost from the iPSC between expansion passages. With the exception of the genomic recombination risk, SeV RNA method encounters the same concerns of episomal system for the clinical application, that is, the passive clearance of RF and false negative results. The mRNA method has been successfully applied in iPSC field, achieving high efficiency and rapid kinetics, without risk of accidental insertional mutagenesis and without the need for multiple passages to clear residual vector traces (Figure 1(b(iii))). Indeed, once transfection of RF is completed, ectopic expression in the cells soon ceases thanks to the rapid degradation of mRNA in the cytoplasm. Synthetic mRNA delivery to cells can occur by electroporation allowing diffusion into the cytoplasm by creating pores in the cell membrane [48] and by complexing the RNA with cationic vehicles permitting internalization by endocytosis after the linkage to the negatively charged cell membrane [49]. Moreover, parallel to mRNA transfect other RNAs (siRNA, miRNA, and long noncoding RNA) can be codelivered with the same method, increasing the possibilities to control reprogramming and differentiation by supplying growth factors, cytokines, and small molecules in culture media [50] (Figure 1(b(iv))).

### 2.3. Xeno-Free iPSC

Another important safety-related issue to translate iPSC into the clinical setting is the need to reduce or eliminate the use of animal-derived materials, establishing xeno-free conditions for both iPSC derivation and expansion (Figure 1, right column).

All the initial culturing techniques for hiPSC utilized mouse embryonic fibroblast feeder cells and media containing other xeno-contaminated reagents, inheriting protocols developed for hESC cells over the last decade. The mouse feeder cell system bears in itself the risk of transmission of nonhuman pathogens to humans as well as immunological issues of rejection triggered by nonhuman antigens [50]. To overcome these obstacles, several protocols have been attempted. Almost all the approaches are based on media optimization toward xeno-free conditions and on the use of extracellular matrix- (ECM-) based feeder-independent culture system [50], substituting the routine system that includes bovine serum albumin (BSA) on Matrigel. Various matrices can be used to replace feeder cells, such as Matrigel, CELLstart, recombinant proteins, and synthetic polymers. Xeno-free media recently developed include TeSR2 and Essential E8 medium [39].

The former, developed by Sun et al. [51] for hESC culturing, is characterized by the complete absence of animal proteins and the inclusion of human serum albumin and human sourced matrix proteins. However, the prohibitively expensive costs of these media make their use not applicable for routine use. Additionally, the high variability of human serum albumin from batch to batch can impact the reprogramming results. When it was clarified that the need of albumin in ES and iPSC media is strictly linked to prevent the toxicity of another component, *β*-mercaptoethanol (BME), contained in the media, and is no longer necessary when BME is removed, a new medium was proposed, defined as E8 (eight components, including the DMEM/F12) [52]. Additionally, surfaces that efficiently support derivation and maintenance of hESC and iPSC were added such as laminin, vitronectin, and fibronectin purified from human plasma, or pericellular matrix of decidua-derived mesenchymal stem cells [52]. Several vitronectin variants were tested and in particular VTN-NC and VTN-N resulted to be efficient [40]. Nakagawa et al. [38] reported that recombinant laminin-511 E8 fragments are useful matrices for maintaining hESCs and footprint-free hiPSCs when used in combination with the StemFitTM medium, completely xeno-free. Their study showed that the Ff-hiPSCs established under footprint-free and xeno-free conditions from several types of somatic cells are similar to the hiPSCs established using the conventional system with feeders, showing equivalent growth and differentiation potential.

### 2.4. hiPSC Differentiation

**Figure 2:**
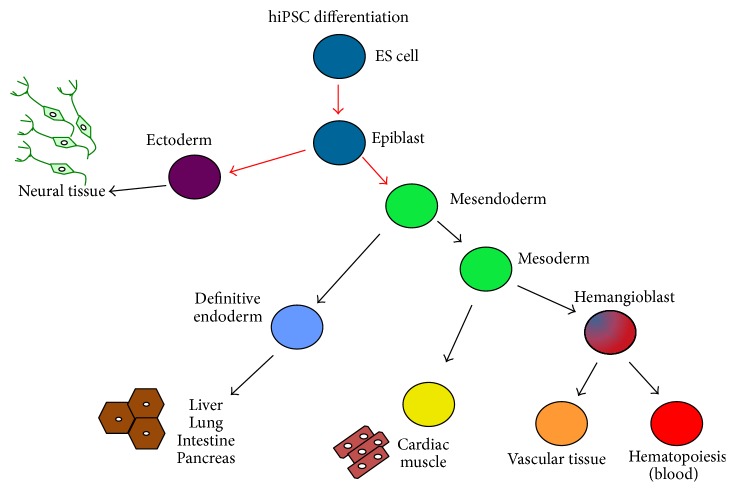
Human iPSC can be differentiated into all cell lineages.

hiPSC, obtained with the methods above, can be differentiated into all cell lineages as shown in Figure 2. Detailed protocols on how to differentiate footprint-free hiPSC were previously reported [16, 53].

## 3. Results

### 3.1. iPSC and Ischemic Stroke

Ischemic stroke, still causing high disability and mortality, prompted the investigation of therapeutic approaches other than thrombolytic therapy and/or percutaneous intravascular interventions [54]. iPSCs have emerged as a promising tool for cell replacement in ischemic brain injuries. At least 4 synergistic mechanisms have been proposed to account for the beneficial effect of stem cells on experimental stroke: neuroprotection, neurogenesis, modulation of the immune response, and angiogenesis. The first [55] occurs by secretion of neuroprotective cytokines such as VEGF and NGF and neurotrophins and by causing paracrine effects, increasing dendritic plasticity and axonal rewriting. Endogenous neurogenesis [56] has been shown by increased number of cells expressing the early neuronal lineage marker Dcx in murine models. Modulation of immune and inflammatory response [57] is achieved by reducing the main inflammatory regulators in focal brain tissue, such as microglia, by inducing the downregulation of some inflammatory regulators, such as TNF-alpha, IL-6, and leptin receptors. Finally, angiogenesis [58] is stimulated with formation of brain microvessels and functional recovery has been demonstrated in peri-infarct regions after stem cell infusion in rat stroke model.

### 3.2. iPSC and SCI

SCI can be caused by a variety of factors, such as trauma, ischemia, and iatrogenic injury, resulting in sensory and motor dysfunctions. SCI [59] is the consequence of the primary irreversible damage caused by direct mechanical insult and the secondary injuries of trauma as inflammatory/immune response, cell necrosis and/or apoptosis, excitotoxins, oxygen free radical, ionic imbalance, and axon reaction. The therapeutic effects of iPSC in SCI can affect multiple mechanisms [60], such as the reconstruction of neural synaptic connections by neural cells derived by iPSC, axons remyelination by oligodendrocytes, and the neuroprotection due to neurotrophic factors secreted by neural cells. In mouse SCI model, data show that treating the damage with iPSC could restore the impaired function through these mechanisms [61]. Mouse iPSC-derived NPC transplanted into nonobese diabetic-severe combined immunodeficiency (NOD-SCID) mice's spinal cord 9 days after SCI differentiated into all three neural lineages did not give rise to teratoma and showed their neural differentiation capacity, participating in remyelination and inducing the axonal regrowth and promoting motor functional recovery [62]. Thus, iPSC clone-derived NPC may be a promising cell source for future transplantation therapy in SCI.

### 3.3. iPSC and Neurodegenerative Disease Modelling

Patient-specific iPSCs provide the unprecedented opportunity to study insights and potentially develop therapeutic options for neurodegenerative diseases, up to date difficult to target due to lack of experimental models. The generation of cell models of diseases is based on the differentiation of disease-specific iPSC into cell types relevant to the diseases [63]. The characterization of iPSC from patient-specific fibroblasts has been reported [64].

Table 1 summarizes the CNS disease-specific iPSCs that have been derived. Most diseases in which the phenotype could be recapitulated were congenital and paediatric disorders [63].

### 3.4. iPSC and Adrenoleukodystrophy Modelling

Jang et al. [24] generated X-linked adrenoleukodystrophy (ALD) iPSC, for both childhood cerebral ALD (CCALD) and adrenomyeloneuropathy (AMN). Both CCALD and AMN iPSC normally differentiated into oligodendrocytes, the cell type primarily affected in the X-linked ALD brain, indicating no developmental defect due to the ABCD1 mutations. Although low in X-ALD iPSC, very long chain fatty acid (VLCFA) level was significantly increased after oligodendrocyte differentiation. VLCFA accumulation was much higher in CCALD oligodendrocytes than AMN, indicating that the severe clinical manifestations in CCALD might be associated with abnormal VLCFA accumulation in oligodendrocytes. Furthermore, the abnormal accumulation of VLCFA in the X-ALD oligodendrocytes can be reduced by the upregulated ABCD2 gene expression after treatment with lovastatin or 4-phenylbutyrate. X-ALD iPSC model recapitulates the key events of disease pathophysiology, as VLCFA accumulation in oligodendrocytes, and allows for early diagnosis of the disease subtypes. X-ALD oligodendrocytes can be a useful cell model system to develop new therapeutics for treating X-ALD.

### 3.5. iPSC and Rett Syndrome Modelling

Using Rett syndrome (RTT) as an autism spectrum disorders genetic model, Marchetto et al. [28] developed a culture system using iPSC from RTT patients' fibroblasts, generating functional neurons. Neurons derived from RTT-iPSC had fewer synapses, reduced spine density, smaller soma size, altered calcium signalling, and electrophysiological defects. Finally, they used RTT neurons to test the effects of drugs in rescuing synaptic defects. Their model recapitulates early stages of a human neurodevelopmental disease.

### 3.6. iPSC and Familial Dysautonomia Modelling

Familial dysautonomia (FD) is a rare but fatal peripheral neuropathy, characterized by the depletion of autonomic and sensory neurons and caused by a point mutation in the* IKBKAP* gene, involved in transcriptional elongation. Lee et al. [27] elaborated the patient-specific FD-iPSCs and evidenced tissue-specific missplicing of* IKBKAP in vitro* by performing gene expression analysis in purified FD-iPSC-derived lineages. Patient-specific neural crest precursors express particularly low levels of normal* IKBKAP* transcript, as a mechanism for disease specificity. They also validated the potency of candidate drugs in reversing aberrant splicing and ameliorating neuronal differentiation. Finally, Koch et al. [30] illustrate that iPSCs enable the study of aberrant protein processing associated with late-onset neurodegenerative disorders in patient-specific neurons in Machado-Joseph disease model.

### 3.7. iPSCs as Gene Therapy Vectors for Brain Tumors

High grade gliomas (HGG), the most common primary brain tumors, remain a clinical challenge with an average life expectancy of 14 months for the most aggressive type after the best surgical, radiation, and chemotherapy treatments [65] due to the tumors' ability to diffusely invade and infiltrate the brain parenchyma. This coupled with the inability of most therapeutic compounds to penetrate the brain due to the blood-brain barrier raises the need to develop vectors that can infiltrate the brain in a fashion similar to glioma tumor cells delivering proapoptotic genes that spare normal parenchyma. Stem cells (SC) seem to be a logical choice as they maintain migratory capacity after transplant into the brain [66].  Table 2 summarizes the SC used as vectors to deliver specific therapeutic agents for HGG. Thus far, three types of SC have been tested as vehicle for therapeutic agents in brain tumors: ESC, mesenchymal SC (MSC), and NPC. Each strategy has specific advantages and disadvantages. ESC can be permanently and genetically modified using homologous recombination [67], but their use is held back by ethical and regulatory issues. NPC are the only SC native to the brain [68]; they have tumor tropism and infiltrative capacity across the blood-brain barrier; however, they are difficult to harvest and have risk of dedifferentiation with potential for tumorigenesis. MSC are easily obtainable from bone marrow and peripheral tissues or blood cells, but a major limitation is safety, due to the risk of promoting the growth potential of HGG cells [69]. Our published work shows that mESC-derived astrocytes maintain migration capacity after implant into the brain and in the presence of brain tumors they “home” within and around it [70]. We have also shown that a proapoptotic gene can be inserted prior to ECS differentiation into astrocytes downstream to a tetracycline inducible promoter “tet-on” to regulate its expression with administration of doxycycline (Dox) [71]. Additionally, we have shown the proapoptotic effects of the mECS-derived astrocytes expressed gene* in vitro* and* in vivo* [72, 73]. Whereas most of the work using stem cells as vector is done on experimental models, there is a current FDA-approved phase 1 clinical trial using NPC engineered to convert the 5FU prodrug into active chemotherapy [74]. However, there are at least 4 significant limitations to this vector: (1) NPC are difficult to obtain and must be derived from fetal brain raising technical and ethical questions; (2) NPC are not fully differentiated and therefore are potentially tumorigenic; (3) viral vectors, used to engineer NPC, cause significant risk of insertional mutagenesis; (4) NPC are not autologous requiring potential immunosuppressive therapy. This prompted us to explore other vectors, such as iPSC.

**Figure 3:**
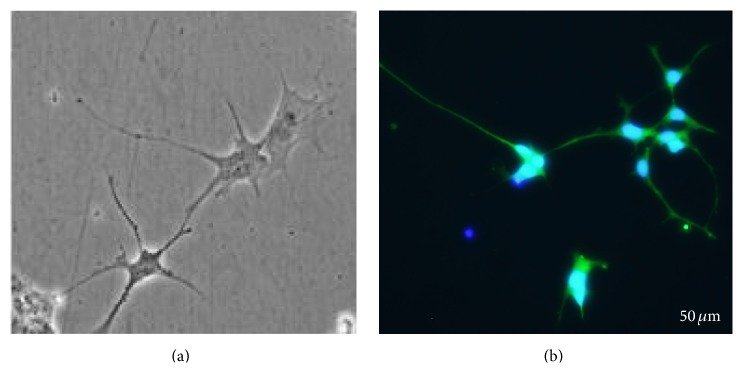
Microphotographs of footprint-free iPSC-derived astrocytes. (a) Phase contrast and (b) immunocytochemistry for GFAP 9 days after MACS sorting of mRNA iPSC-derived astrocytes.

**Figure 4:**
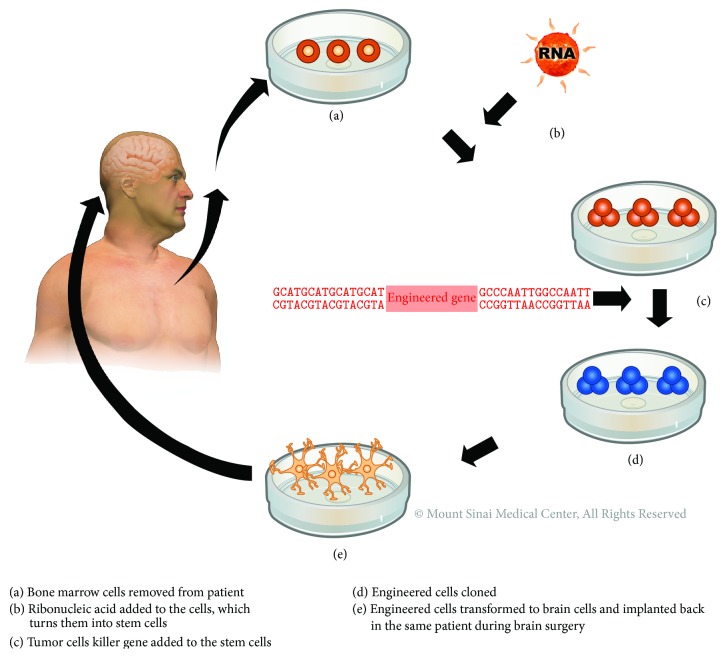
Personalized medicine using patient-specific iPSC. Diagrammatic summary of reprogramming patient-specific cells into footprint-free hiPSC, engineering their DNA to carry proapoptotic genes, differentiating them into astrocytes, and reimplanting them at the time of surgery for brain tumor recurrence. (a) Dermal fibroblast cells obtained from patient. (b) Ribonucleic acid (RNA) added to cells, which turns them into stem cells. (c) Tumor cells killer gene added to stem cells. (d) Engineered cells cloned. (e) Engineered cells transformed to brain cells, astrocytes, and implanted back in the same patient at the time of surgical resection for recurrent tumor.

We have shown that we can differentiate astrocyte from iPSC in similar fashion to those obtained from ESC [21]. Recently, we have also shown that we can differentiate a pure population of footprint-free iPSC-derived astrocytes (Figure 3), which does not cause teratogenicity after implant into the brain [53]. We therefore propose that patient-specific cells can be reprogrammed into “footprint-free” hiPSC, their DNA engineered to carry proapoptotic genes, and then be differentiated into astrocytes and reimplanted in the same patient at the time of surgery for brain tumor recurrence (Figure 4). As discussed below, the ability to translate these exciting data to the clinical setting is still halted by technical obstacles, cost-effectiveness, and scalability.

## 4. Discussion

Human SC represent important cell resources and hold high promise for disease modelling, cell-based therapies, and drug and pharmaceutical applications [75]. iPSCs are the most appealing among SC due to the recent advances in reprogramming footprint-free and xeno-free iPSC [53]. They are a promising platform to pave the way for personalized medicine as they can be differentiated from the same patient to study his/her disease and/or response to new drugs and/or delivered back carrying proapoptotic genes/drugs. Current limitations, however, are still halting the translational use of hiPSC and need additional technical improvements. These are limited to not only the reprograming process, such as genetic/epigenetic abnormalities and immunogenicity, but also cost and labor of reprogramming process.

Genetic and epigenetic abnormalities may be reduced during reprogramming by improving efficiency to a level where iPSC could be derived without colony picking and colonial expansion, because the low efficiency and slow kinetics of iPSCs generation may give rise to the activation of cell growth pathways and suppression of tumor suppressor pathways. Therefore, using epigenetic small molecules to improve reprogramming efficiency could represent the key to ensure greater iPSCs safety. Reprogramming with mRNA could be highly immunogenic [76], since human cells have antiviral defence pathways triggered by exogenous RNA. These pathways can activate the suppression of translation, the degradation of foreign transcripts, and the priming of cytostatic and apoptotic pathways. To avoid such immunogenic response, several strategies have been tested, such as the incorporation of modified nucleobases (pseudouridine) into synthetic transcripts [77] or the supplementation of cell media with an extracellular decoy receptor for type I interferons [78] that blunt immune responses to infection.

A major obstacle in using iPSCs for clinical application resides in the risk of genomic modification when they are derived with viral transgenes, but the generation of “footprint-free” iPSC-derived astrocytes represents a promising innovation. Nonetheless, some drawbacks still exist even with mRNA reprogramming. First, certain cell types, including blood cells, are difficult to transfect [79]. Secondly, the approach works robustly if mRNA is transfected at frequent intervals to yield a steady state of protein expression over time. Cationic transfection reagents come to aid since they are well tolerated on repeat administration, while electroporation procedures are less feasible.

To achieve their full clinical and commercial potential, significant challenges must be overcome in order to produce iPSC-derived cells at commercially relevant scale. These include operational performances, economics, quality control and compliance, safety, and flexibility. Recent innovations in integrated bioprocesses design are helpful in improving hiPSC expansion. These include planar and three-dimensional culture systems. In particular, planar processing platforms are important for the production of autologous and patient-specific hiPSC-derived cells that necessitate a scale-out rather than a scale-up process [80]. Additional improvements are needed in the differentiation processes, including planar strategies and bioreactor-based systems. Finally, shorter reprogramming process and strategies to rapidly induce iPSC need to be developed as well as media to improve iPSC efficiency without causing any aberrations of reprogrammed cells [81].

## 5. Conclusion

iPSCs provide a novel platform for CNS regenerative medicine, neurodegenerative disease modelling, pharmaceutical testing, and brain tumor treatments with a personalized medicine paradigm. The unique properties of iPSCs to self-renew and to differentiate into cells of three germ layers make them an invaluable tool for the present and the future of most neurologic disorders. Technical improvements in reprogramming with high efficiency induction systems and virus-free and integration-free strategies have greatly advanced iPSC therapeutic potentials. Additional efforts focused on refining reprogramming approaches will further enhance their clinical applications. Current efforts to reduce labour and cost are also instrumental for the integration of iPSC and iPSC-derived cells in the clinical setting.

## Figures and Tables

**Table 1:** Neurodegenerative specific iPSC for disease modelling.

CNS disease	Genetic defect	Phenotype
Adrenoleukodystrophy [24]	ABCD1	Increased level of VLCFA in oligodendrocytes

Alzheimer's disease [25]	Presenilin 1 Presenilin 2 APP duplication	Increased amyloid *β* (A*β*) secretion Increased A*β*40 production Increased phosphor-tau and GSK-3*β* activity

Amyotrophic lateral sclerosis [3]	SOD1, VAPB, and TDP43	Decreased VAPB in motor neurons Elevated levels of TDP43 protein

Huntington's disease [26]	CAG repeat expansion in HTT gene	Enhanced caspase activity upon growth factor deprivation

Familial dysautonomia [27]	IKBKAP	Decreased expression of genes involved in neurogenesis and neural differentiation

Parkinson's disease [3]	LRRK2, PINK1, and SNCA	Impaired mitochondrial function in PINK1-mutated dopaminergic neurons Increased sensitivity to oxidative stress in LRRK2 and SNCA-mutant neurons

Rett syndrome [28]	MeCP2 CDKL5	MeCP2: neuronal maturation defects, decreased synapse number CDKL5: aberrant dendritic spines

Spinal muscular atrophy [29]	SMN1	Decreased size, number, and survival of motor neurons

Machado-Joseph disease [30]	MJD1 (ATXN3)	Excitation-induced ataxin-3 aggregation in differentiated neurons

Schizophrenia [31]	Multifactorial	Reduced neuronal connectivity, increased consumption in extramitochondrial oxygen, and elevated levels of ROS

VLCFA: very long chain fatty acid; ROS: reactive oxygen species.

**Table 2:** Therapeutic agents delivered by SC for the treatment of HGG.

Agent delivered	Type of stem cells		
	ESC	NSC	MSC
Cytokines	Mda-7/IL24, TRAIL	IL-4, IL-12, IL-23, TRAIL +/− BMZ, and S-TRAIL +/− MIR/TMZ	IL-2, IL-12, IL-18, INF*α*, INF-*β*, and TRAIL +/− PI3KI

Enzyme/prodrug		Tk/GCV, CD/5FC +/− IFN*β*	Tk/GCV

Viral particles		Mutant HSV-1, CRAd-survivin	CRAd-survivin, CRAd-CXCR4, and CRAd-Rb

Metalloproteinases		PEX	

Antibodies			EGFRvIII

Nanoparticles			Ferrociphenol lipid
